# Transcriptome Profiling Revealed Multiple *rquA* Genes in the Species of *Spirostomum* (Protozoa: Ciliophora: Heterotrichea)

**DOI:** 10.3389/fmicb.2020.574285

**Published:** 2021-01-05

**Authors:** Irum Mukhtar, Siyi Wu, Shurong Wei, Ruanni Chen, Yunying Cheng, Chen Liang, Jianming Chen

**Affiliations:** Institute of Oceanography, Minjiang University, Fuzhou, China

**Keywords:** ciliates, RNA-seq, anaerobic respiration, heterotricheaa, *Spirostomum*, *Blepharisma*, *Stentor*, *rquA* genes

## Abstract

Adaptation to life at different oxygen tensions plays a role in protozoan ecology and controls the distribution of different species in anoxic habitats. The ciliate genus *Spirostomum* inhabiting fresh or low salinity water globally where these species are considered as bioindicators. Under anaerobic or low oxygen conditions, the rhodoquinol-dependent pathway has been reported in the species from the class Heterotrichea. With the help of RNA sequencing (RNAseq) data, *Spirostomum* spp., are suitable for deep molecular investigations on *rquA* for rhodoquinone (RQ) biosynthesis. In this study, *Spirostomum ambiguum*, *Spirostomum subtilis*, and *Spirostomum teres* collected from densely vegetated freshwater habitat in Fuzhou, China, explored the evidence of *rquA*. Based on transcriptome analysis, two to three RquA proteins were identified in *S. ambiguum*, *S. teres*, and *S. subtilis*, respectively. The presence of a key Motif-I of RquA and mitochondrial targeting signals (MTS), also confirmed the identity of these as RquA. Furthermore, *Spirostomum* RquA proteins could be sorted into two groups based on their conserved amino acid (CAA) residues. Phylogenetic analysis also exhibited RquA division into two subclades contained RquA1 and RquA2/RquA3 and supports two to three paralogs of *rquA* genes in the genomes *Spirostomum* spp. Additional transcriptomes and genomes analysis of *Blepharisma* spp., and *Stentor* spp., respectively, also revealed at least two paralogs of *rquA* in members of the class Heterotrichea. The present study provides evidence for the presence of RquA and rhodoquinol dependent fumarate reduction pathway in *Spirostomum* species potentially use to respire in the oxygen-depleted habitats and two to three diverse *rquA* genes.

## Introduction

The *Spirostomum* Ehrenberg, 1834 is one of the genera in the class Heterotrichea and mostly found in fresh and brackish water habitats. The species of this genus are extensively studied as the model bioindicator organisms for environmental, ecological, microbiological, and ecotoxicological analyses (Madoni et al., 1992; Berger et al., 1997; Madoni, 2000; Berger and Foissner, 2003). Generally *Spirostomum* spp., are elongated, slightly flattened, unicellular ciliated bodies that contract in a spiral way (Boscaro et al., 2014; Fernandes et al., 2016; Shazib et al., 2016; Chen et al., 2017). Like many protist groups, *Spirostomum* spp., are usually found in the sediment deposits of water bodies, and thrive well in these habitats. Aquatic sediments are always anoxic at some depths due to the debris accumulation, biofilms, and low oxygen transport. The anoxic zone may reach or even rise above the surface in productive shallow-water sediments and *Spirostomum* spp., can boom easily in such type of freshwater environments due to their omnivorous/detritivorous feeding mode of nutrition (Song et al., 2009; Méndez-Sánchez et al., 2018; Hu et al., 2019).

Many ciliates are well known to be able to survive under anaerobic conditions by adaptation of different ways of energy metabolism (Yarlett et al., 1981; Finlay et al., 1983; Esteban et al., 2009). Despite their significant role in the different waterbodies, *Spirostomum* spp., experience hypoxia or even anoxia every day while living in shallow water sediments. In anaerobic respiration pathway, rhodoquinone (RQ) is an essential cofactor in bacteria and many eukaryotes (Stairs et al., 2018) that utilize a fumarate reductase pathway. Based on chemical structure, RQ is also known as aminoquinone similar to ubiquinone (Q = coenzyme Q: a polyprenylated benzoquinone) which needed in the aerobic respiratory chain (Brajcich et al., 2010; Lonjers et al., 2012). However deep information on the gene(s) involve in RQ synthesis from ciliate species is mostly unavailable so far, with only a limited record (Stairs et al., 2018). Transcriptome sequencing and bioinformatics analysis can efficiently evaluate genes related to different processes in eukaryotes. The whole transcriptome profiling can reveal genes that are differentially expressed under different habitats. The presence of *rquA* gene(s) in some protists reflect the anaerobic activity and could become important research areas for understanding and explore new orthologous of these genes. Recently two *Spirostomum* species collected from low oxygen freshwater habitat have been reported for *rquA* (Hines et al., 2018). The presence of this anaerobic pathway related gene makes members of *Spirostomum* as well as other species in the class Heterotrichea ideal candidates to investigate it in detail.

To get insight into the diversity of *rquA* and genetic linkage within genus *Spirostomum*, transcriptomic data of more species within this genus is needed. In this study, RNA sequencing data of three *Spirostomum* species (*Spirostomum ambiguum*, *Spirostomum subtilis*, and *Spirostomum teres*) was specifically targeted, collected from sediment layers in a freshwater pond habitat in Fuzhou, China. Transcriptome data generated in this study contain an important and improved understanding of multiple *rquA* in the *Spirostomum* genus and the whole suborder Heterotrichea.

## Materials and Methods

### Sampling and Ciliate Isolation

*Spirostomum* species samples were collected from the bank of a freshwater pond located in Fujian Agriculture and Forestry University, Fuzhou city, China (Supplementary Figure S1). Previously, neither ciliate biodiversity nor *Spirostomum* species have been explored and reported from this location. The pond bank habitat is densely packed with plants and rich in organic sediment. The point sampled in the pond had a depth of less than 30 cm. Oxygen levels were very low (<5%).

The samples were collected during the mild period in September 2019; though, *S. ambiguum* and *S. subtilis* kept thriving in collected samples even in colder December 2019 and January 2020 and could also recover from the collection site. Pond water samples were transported in 1000 mL containers and examined within 3–4 h of collection. Water samples were directly poured into 5-cm petri dishes for ciliates isolation. The sampling site contains a diverse and exciting assembly of the ciliate community. The community was dominated by *Loxodes rostrum* and *Stentor* species.

*Spirostomum* species cells were hand-picked from samples under a dissecting microscope using a micropipette, identified according to morphological criteria, using detailed live observation. To set the monoclonal culture, the individual cell was washed in sterile distilled water and kept in rice grains supplemented filtered water medium in 5 cm petri dish for 15 days at 28 °C, before molecular identification and transcriptome amplification. Rice grains were periodically added to stimulate the growth of prey bacteria in the water medium.

### Ribosomal Genes Amplification and Sequencing

In order to ensure the identity of the individual *Spirostomum* cultures, cell from each clonal culture was used for molecular identification using different primer sets. For molecular confirmation, one or more cells from individual clonal culture were collected and washed at least three times to remove contaminants and transferred into PCR microtubes with 3 μL of sterile water, 1 μL of each primer and 45 μL of PCR mix to make final reaction volume of 50 μL per sample. To amplify the small subunit (SSU) rDNA gene forward EukA (AACCTGGTTGATCCTGCCAG) and reverse EukB (CACTTGGACGTCTTCCTAGT) primers (Medlin et al., 1988) were used. PCR amplifications were performed using a Super PCR Mix (Tsingke, China) under the following conditions: 1 cycle (2 min at 98°C); 34 cycles (10 s at 98°C, 20 s at 57°C, 30 s at 72°C) and 1 cycle (2 min at 72°C). ITS region (ITS1-5.8S-ITS2) was amplified using ITS-F (GTTCCCCTTGAACGAGGAATTC) and ITS-R (TACTGATATGCTTAAGTTCAGCGG) primers (Fernandes et al., 2016), with the following cycling parameters: 1 cycle (2 min at 98°C); 34 cycles (10 s at 98°C, 20 s at 56°C, 30 s at 72°C) and 1 cycle (2 min at 72°C). The D1D2 fragment of the LSU (28S) rDNA gene was amplified with D1D2-fwd1 (AGCGGGAGGAAAAGAAACT) and D1D2-rev2 (ACGATCGATTTGCACGTCAG) primers (Stoeck et al., 2014), with the following cycling conditions; 1 cycle (2 min at 98°C); 34 cycles (10 s at 98°C, 20 s at 54°C, 30 s at 72°C) and 1 cycle (2 min at 72°C). The size of all amplified DNA fragments were confirmed by electrophoresing in 1% agarose gel and 1× TAE buffer at 120 V for 30 min. PCR products were visualized with the 4S-Red Plus Nucleic Acid Stain (Sangon Biotech, China) and UV transillumination. Later, PCR products were sequenced (Sangon Biotech, China), using respective PCR primers.

### Sequence Processing and Phylogenetic Analysis

The obtained sequence files from three species (*S. ambiguum*, *S. subtilis*, and *S. teres*) were checked for the quality and assembled using Chromas 2.6.6 (Technelysium Pty, Ltd.) and Clone Manager suite 7 (Sci Ed Software LLC., United States), respectively. To infer phylogenetic analysis, the reference sequences of SSU rDNA of other *Spirostomum* spp., as well as closely related genera were obtained from NCBI and SILVA database according to literature (Shazib et al., 2014, 2019; Yan et al., 2016; Hines et al., 2018; Chi et al., 2020). As an outgroup support, SSU sequences were selected from *Loxodes* and *Remanella*. All sequences were aligned and analyzed on the web server Phylogeny.fr^1^ using “one click” option. On this server site, the MUSCLE (Edgar, 2004; Dereeper et al., 2008, 2010) and Maximum likelihood (ML) method using HKY85 model with default parameters, were used for sequence alignment and phylogenetic tree construction, respectively.

To show monophyletic group of heterotrichs species in Supplementary Figure S2, the SSU rDNA nucleotide sequences were aligned using Clustal W (Thompson et al., 1994) and phylogenetic tree was constructed using Neighbor-Joining (NJ) method with the Kimura-2 parameter model in MEGA version 6.0 (Tamura et al., 2013).

### cDNA Generation and Transcriptome Assembly

Nearly 1500 cells of each species of *Spirostomum* were handpicked separately using pipettes and collected into eppendorf tubes (1.5 mL), centrifuged at 3000 rpm and room temperature for the 20 s and removed the supernatant. Cells were washed gently by added filtered (0.2 μm) *in situ* water, centrifuged (3000 rpm for the 20 s), and decanted the supernatant. Cells washing was repeated twice before being placed in a minimal volume of nuclease-free water in a microcentrifuge tube, and quickly frozen first in liquid nitrogen followed by dry ice and sent to Novogen, Beijing, China for RNA extraction and transcriptome.

Total RNA was extracted from *Spirostomum* cells using RNeasy Plus Mini Kit (QIAGEN, China) in accordance with the manufacture’s instructions. For the removal of residual DNA, extracted RNA was treated with DNase I (Takara, Japan) for 40 min at 37°C and was quantified using Nanodrop 2000 (Thermo Fisher Scientific, United States). The RNA from three biological samples (1.5 μg RNA @ per sample) of each species were pooled together for library construction. With the help of NEBNext^®^ Ultra^TM^ RNA Library Prep Kit for Illumina^®^ (NEB, United States) according to manufacturer’s recommendations, sequencing libraries were generated and index codes were added to attribute sequences to each sample. The mRNA with poly (A) was isolated from total RNA using poly-Toligo-attached magnetic beads. The NEBNext First Strand Synthesis Reaction Buffer (5×) was used to cut mRNA randomly, and First strand cDNA was synthesized using these fragments as templates along with random hexamer primer and M-MuLV Reverse Transcriptase (RNase H) and purified using AMPure XP beads (Beckman, United States). For Second strand cDNA synthesis, DNA Polymerase I and RNase H were used. Remaining overhangs were converted into blunt ends via exonuclease/polymerase activities. This was followed by end repair, adenylation, and NEBNext Adaptor ligation with hairpin loop structure of purified cDNA. For PCR amplification, cDNA fragments of preferentially 250∼300 bp in length were selected as templates. The size-selected, adaptor-ligated cDNA were treated with 3 μL USER Enzyme (NEB, United States) at 37°C for 15 min followed by 5 min at 95°C before PCR. The Phusion High-Fidelity DNA polymerase, Universal PCR primers, and Index (X) Primer, were used for PCR. Amplified products were purified using AMPure XP system and library quality was assessed on the Agilent Bioanalyzer 2100 system. The clustering of the index-coded samples was performed on a cBot Cluster Generation System using TruSeq PE Cluster Kit v3-cBot-HS (Illumia) according to the manufacturer’s instructions. After cluster generation, the library preparations were sequenced on an Illumina Hiseq platform and paired-end reads were generated.

### Data Quality Assessment, Assembly, and Clustering

The base quality of the obtained raw data was evaluated using Qphred software. The reads of low-quality such as reads containing adaptor or primer sequences, reads with undetermined bases proportion greater than 10%, reads with more than 50% bases proportion with Qphred ≤ 20 and so on were discarded, and the remaining clean reads were kept for assembly. According to Grabherr et al. (2011), Trinity (Trinity-v2.5.1) software was used for transcriptome assembly and assembled TRINITY. fasta were finally obtained.

To obtain non-redundant reads and gene-level counts from each sample, Corset v.1.05 (Davidson and Oshlack, 2014) was utilized to hierarchically cluster the transcripts. The longest Cluster sequence was used for further analysis (Corset website^2^).

### Identification of Anaerobic Respiration Pathway and RquA Sequences

Transcriptomes of three *Spirostomum* species were explored for previously reported anaerobic respiration related proteins in eukaryotes (Müller et al., 2012; Stairs et al., 2015). To confirm the presence of hydrogenosomes, key enzymes, i.e., FeFe-hydrogenase, pyruvate:ferredoxin oxidoreductase (PFO = PFOR = pyruvate synthase) and maturase proteins (HydE, HydF, and HydG) were used as queries against the transcriptomes of the *Spirostomum* spp. Moreover, the sequences of NuoE and NuoF and RquA, involved in RQ biosynthesis in ciliates and other eukaryotes, were searched against the transcriptomes of *Spirostomum* spp., to confirm the hydrogenase and RQ biosynthesis pathway (Stairs et al., 2018).

As pyruvate formate-lyase (PFL) is an essential enzyme in anaerobic metabolism and responsible for converting pyruvate and coenzyme A (CoA) into formate and acetyl CoA. We used tblastn search with PLF and PFLa (PLF activator) sequences from protists i.e., [*Mastigamoeba balamuthi* (ADM53419, AQM57589); *Stygiella incarcerate* (ANM86870)] and algae [*Chlorella sorokiniana* (PRW57093); *Chlamydomonas reinhardtii* (gi:92084842, 57021069); *Fistulifera solaris* (GAX24722); *Monoraphidium neglectum* (KIZ05615)], as queries to detect PFL activity.

Within ciliates, we also searched *rquA* homologs in the genomes of *Entodinium caudatum* (PRJNA380643), *Euplotes focardii* (PRJNA329414), *Oxytricha trifallax* (AMCR00000000.2), *Paramecium caudatum* (PRJNA246569), *Paramecium sexaurelia* (PRJNA252373), *Paramecium tetraurelia* (PRJNA19409), *Paraurostyla* sp. (PRJNA274287), *Pseudokeronopsis carnea* (PRJNA507672), *Pseudocohnilembus persalinus* (PRJNA275811), *Stentor roeselii* (PRJNA507905), *Stylonychia lemnae* (PRJEB5807), *Strombidium stylifer* (PRJNA599298), *Uroleptopsis citrina* (LXJT00000000.1), *Urostyla* sp. (PRJNA274288), *Tetrahymena borealis* (PRJNA51575), *Thorichthys elliotti* (PRJNA51573) available on Genbank. Due to similar point of isolation, additional transcriptomic data of *Blepharisma musculus* (SRR12647629) and *Blepharisma undulans* (SRR12647630) were also explored for *rquA* in this study.

### Confirmation of *rquA* Genes in *Spirostomum* Species

Presence or absence of *rquA* genes in *S. ambiguum*, *S. subtilis*, and *S. teres* were confirmed using specific primers (Supplementary Table S1), based on transcriptomic datasets. PCR was performed in volumes of 50 mL Super PCR Mix (Tsingke, China), using 34 cycles and an initial melting step of the genomic DNA at 98°C for 2 min. Amplified products were sequenced (Sangon Biotech, China), using respective *rquA* primers. For identity and structure of each *rquA* in *Spirostomum* species, multiple sequence alignments of genomic and transcriptomic sequences were performed with Clustal W.

### Lipid Extraction and Detection of RQ

*Spirostomum* spp., *Blepharisma* spp., and bacterial prey cells were collected separately from 15 days old cultures by centrifugation at 3000 rpm for 1 min, respectively. Cell pellet was extracted using a modified method (Drochioiu, 2005) by adding aceton volume @ six times of pellet, vortexed vigorously, and separated by centrifugation at 8000 rpm for 2 min. Supernatants were transferred to a new test tube, and the remaining cell residues were re-suspended into acetone (added @ double the volume of residues) for second extraction, and repeating the procedure. Extracts were pooled, evaporated to dryness, and sent to Xiamen Minxi Technology CO., LTD., Xiamen, China, for the detection of RQ or Q using liquid chromatography (LC) and mass spectrometry (MS).

Chromatography was performed by using a Thermo C18 column (2.1 mm × 150 mm, 5 μm; Thermo U3000, Thermo Fisher Scientific, United States). All UPLC runs using a flow rate of 0.25 mL/min and an injection volume of 5 μL. All injections were performed in duplicate. RQ_10_ and Q_10_ were eluted by using a gradient system containing water with 0.1% formic acid (buffer D) in ddH_2_O and acetonitrile (ACN) with 0.1% formic acid (buffer A). Elution gradient (buffer A–buffer D) method used was as follows; 0–5 min 30% D 70% A; 5–10 min (2% D: 98% A); 10–20 min (2: 98); 20–25 min (2: 98 to 1: 99); 25–35 min (1: 99); 35–40 min (1: 99 to 30: 70); 40–45 min (30:70). Mass spectral analysis was accomplished using the Thermo Scientific Q Exactive (QE) Mass Spectrometer (Thermo Fisher Scientific, Untied States) in W-positive mode with an extended dynamic range. All samples were analyzed for the presence of RQ_10_ and Q_10_ using following conditions: Resolution, 70,000; AGC target, 3e6; Maximum IT, 100 ms; Scan range, 200–1800 m/z; dd-MS2/dd-SIM; Resolution, 17,500; AGC target,1e5; Maximum IT,100 ms, Loop count, 20; Isolation window, 2.0 m/z; (N)CE/stepped nce, 30.

## Results

Three species (*S. ambiguum*, *S. subtilis*, and *S. teres*) of genus *Spirostomum* found thriving in a freshwater pond matched having a characteristic habitat that previously described records (Esteban et al., 2009). Cell densities of ciliates in collected samples were observed up to10 cells per mL. The ciliates kept thriving naturally for more than four months in the samples. Cysts were not observed in the culture of *Spirostomum* spp.; however, there are records of *Spirostomum* species being able to form cysts precursors (Ford, 1986; Hines et al., 2018).

The cells of *S. ambiguum* were very large (1000–4000 μm long; length: width ratio about 9–17), elongated with a cylindrical body, slightly cream in color, and were highly contractile. Living colorless *S. subtilis* cells were 700–1000 μm long (length: width ratio about 14–24) with conspicuous contractile vacuole up to 1/3 of the body length. Fully extended living *S. teres* cell had a rounded posterior end and tapered anterior end. *S. teres* cells were 150–600 (avg. 250–450) μm long and approximately 65 μm in width (length: width ratio about 5–16). Live cells were colorless and moved slowly, gliding over the substrate. A single, conspicuous contractile vacuole was observed at posterior third of the cell.

### Phylogeny of *Spirostomum* Species

**FIGURE 1:**
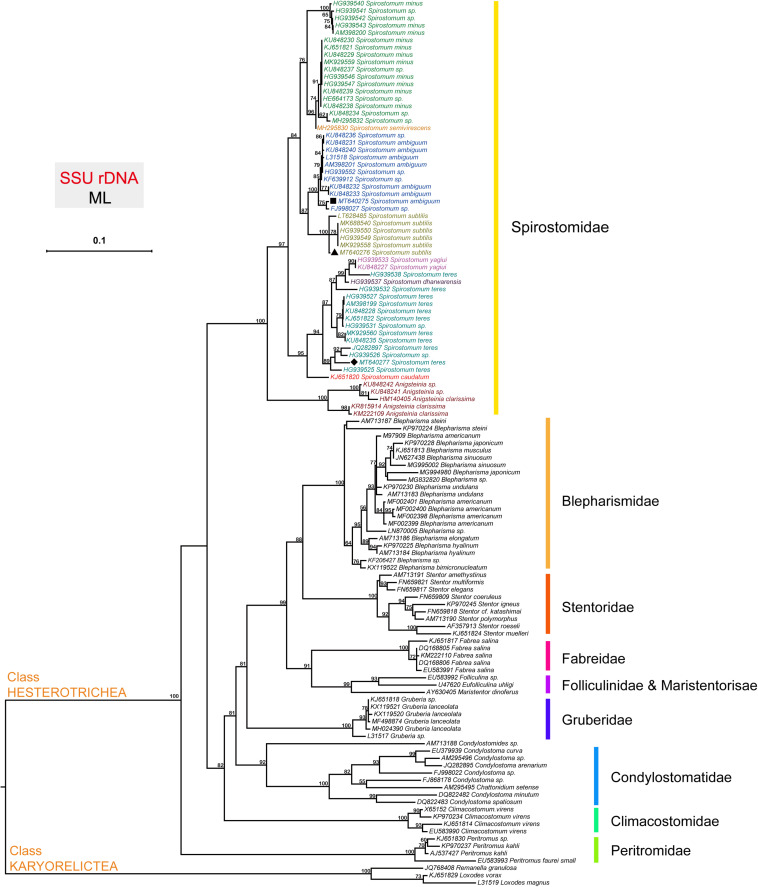
The phylogenetic position of species within the genus *Spirostomum*. The topology of the tree is inferred from SSU sequences using the Maximum likelihood (ML) method according to the similarity of sequences, and the results were obtained with “one click” option in the server http://www.phylogeny.fr. Obtained SSU rDNA sequences from (■) *S. ambiguum* (MT640275), (▲) *S. subtilis* (MT640276), and (◆) *S. teres* (MT640277) in this study are positioned within their expected clade according to literature (Chi et al., 2020).

The topology of the tree based on SSU rDNA sequences showed that *Spirostomum* spp., grouped into various clades as reported in previous studies (Boscaro et al., 2014; Shazib et al., 2014; Hines et al., 2018; Chi et al., 2020). The phylogenetic tree indicates that the newly identified *S. ambiguum* (MT640275), *S. subtilis* (MT640276), and *S. teres* (MT640277) are related to their respective clades with maximum support. The populations of *S. ambiguum* and *Spirostomum* spp., form a strongly supported single clade (Figure 1). The Fuzhou population of *S. subtilis* clusters with reported *S. subtilis* population from Europe, Korea, India, and China with maximum support. In the case of *S. teres*, clade position and species arrangement remained according to previous studies (Hines et al., 2018; Chi et al., 2020). The isolate *S. teres* (HG939538) showed affinity with *Spirostomum dharwarensis* and *Spirostomum yahiui*, while the remaining *S. teres* populations grouped into two other subclades with inconstant support values. Results also showed that the Fuzhou population of *S. teres* (MT640277) is closely related to European and South American populations, while recently reported *S. teres* (MK929560) population from China, positioned with Korean isolate. In this study, high quality SSU (18S) rDNA, ITS and LSU (28S) rDNA sequences with reliable identity to respective species could also be found in transcriptomes of *S. ambiguum*, *S. subtilis*, and *S. teres*. Nucleotides sequences of ITS and 28S are also deposited in GenBank for *S. ambiguum* (MW009119), *S. subtilis* (MW009120), and *S. teres* (MW009121).

### Sequencing and Transcriptome Quality

The *de novo* transcriptome assemblies presented in this study provide a complete information on DNA coding in the three *Spirostomum* spp. Current transcriptomic data opens new horizons and expands opportunities for studies on comparative evolution of class Heterotrichea living in low oxygen habitat. For three transcriptomes (Table 1), 8.07–9.68 Gb sequencing data was generated for *Spirostomum* species in this study. After quality filtering, the *S. ambiguum*, *S. subtilis*, and *S. teres* libraries, each retained ∼80% of their paired-end reads. Excluding sequences below 500 nucleotides, 40,465–42,405 high-quality unigenes (UGs) were assembled with an N50 of 1271–1687 (Table 1). In case of *S. ambiguum* only 31,723 UGs could be annotated as proteins representing 78% of UGs assembly of this species. Whereas 74–77% of *S. teres* and *S. subtilis* UGs could be annotated, respectively, against different data bases. GC content (%) and total clean bases range in transcriptomes of *S. ambiguum*, *S. subtilis*, and *S. teres* are according to previously reported for *Spirostomum semivirescens* (Hines et al., 2018). Raw sequences has been deposited to NCBI Short Read Archive (SRA) database^3^ for *S. ambiguum* (SRX8646480), *S. subtilis* (SRX8646481), and *S. teres* (SRX8646482).

**TABLE 1:** Summary of transcriptome sequencing, assembly and annotation of three *Spirostomum s*pecies (*S. ambiguum*, *S. subtilis*, *S. teres*).

**Description**	***S. ambiguum***	***S. subtilis***	***S. teres***
Total raw reads (M)	61,809,532	66,435,450	54,919,056
Total clean reads (M)	60,246,398	64,555,952	53,806,806
GC content%	56.7	51.79	53.63
Total clean bases (GB)	9.04	9.68	8.07
Total number of transcripts	97,443	57,489	55,992
N50 of transcripts	3043	1507	1314
Mean length of transcripts	1886	1085	992
Total number of unigenes (UGs)	40,465	41,540	42,405
N50 of UGs (bp)	1687	1360	1271
Mean length of UGs (bp)	1167	1019	982
Total UGs annotated	31,723	32,246	31,546
UGs annotated against NR data base	26,567	26,304	25,575
UGs annotated against NT data base	7697	3612	5887
UGs annotated against Swiss-Port data base	20,965	19,387	19,456
UGs annotated against GO data base	25,071	25,222	24,455
UGs annotated against KOG data base	11,994	11,992	10,521
UGs annotated against KO(KEGG) data	8014	9473	6337
UGs annotated against Pfam data base	25,071	25,222	24,455

### Identification and Motif Analysis of RquA

Species of genus *Spirostomum* are often found in oxygen-depleted habitat and require to be able to respire under anoxic conditions. To get a better understanding of anaerobic existence of *Spirostomum* species, we conduct a comprehensive search for anaerobic pathways related proteins in the transcriptomes of *S. ambiguum*, *S. subtilis*, and *S. teres* (Müller et al., 2012; Stairs et al., 2015, 2018). The tblastn search showed the evidence of hydrogenosomes, PFL activity, in *S. ambiguum* and *S. teres* as previously reported in some eukaryotes; however no such clear indication was found in the case of *S. subtilis*. We could also not find any evidence of dissimilatory nitrate reduction in *S. ambiguum*, *S. subtilis*, and *S. teres*. In a recent study hydrogenosomes, PFL activity and dissimilatory nitrate reduction were not identified in the transcriptomes of *Spirostomum* species (Hines et al., 2018).

A tblastn search for the RquA of the bacterium *Rhodospirillum rubrum* (WP_011390975), and ciliate *Spirostomum semivirscens* (GGNT01020088); *Spirostomum* sp., (GGNU01007111) (Hines et al., 2018), could identify at least two distinct hits (TRINITY_DN35127, TRINITY_DN38787) in the transcriptome of *S. ambiguum*. Comparison of deduced partial amino acid sequences of *S.am*-RquA1 (TRINITY_DN35127) and *S.am*-RquA2 (TRINITY_DN38787) revealed 41–45% identity and 57–59% similarity with query sequences of RquA from *R. rubrum* and 63–81% identity and 79–89% similarity with their reported counterparts in *S. semivirscens* and *Spirostomum* sp.

Similarly, three distinct hits (TRINITY_DN8118, TRINITY_DN47766, and TRINITY_DN11041) were found in the transcriptome of *S. subtilis.* Sequences of *S.sb*-RquA1 (TRINITY_DN8118), *S.sb*-RquA2 (TRINITY_DN47766), and *S.sb*-RquA3 (TRINITY_DN11041) showed 40–45% identity and 57–61% similarity with *R. rubrum* (WP_011390975) and 65–80% identity and 81–89% similarity with putative RquA sequences of *S. semivirscens* and *Spirostomum* sp., respectively. In the case of *S. teres*, two potential hits (TRINITY_DN46135, TRINITY_DN5375) were identified as a result of tblastn search in the transcriptome. Identified sequences *S.te*-RquA1 (TRINITY_DN46135) and *S.te*-RquA2 (TRINITY_DN5375) showed 35–46% identity and 54–59% similarity with *R. rubrum* (WP_011390975) and 64–80% identity and 83–89% similarity with the RquA of *S. semivirscens* and *Spirostomum* sp., respectively.

During a detail screening of transcriptomes of *S. semivirscens* (GGNT00000000) and *Spirostomum* sp. (GGNU00000000) deposited in GenBank (Hines et al., 2018), four additional RquA [(in *S. semivirscens*; GGNT01022391, GGNT0106908), (in *Spirostomum* sp.; GNU01008548, GNU01010926)] hits were found. These newly identified RquA in *S. semivirscens* and *Spirostomum* sp., showed a high similarity with RquA1 and RquA2 types of *S. ambiguum*, *S. subtilis*, and *S. teres*. Presence of multiple *rquA* most likely facilitate the adaptation of the free living *Spirostomum* spp., to oxygen-depleted environment.

In this study, we could also identify new *rquA* from transcriptomes and genome of three representatives of the class Heterotrichea i.e., *B. musculus* [SRR12647629; (Trinity_DN13012; Trinity_DN9607)] *B. undulans* [SRR12647630; (Trinity_DN27608, Trinity_DN41429)] and *S. roeselii* (RSEH0100050, RSEH01000112), respectively. However, a genome-wide search showed no indication of *rquA* paralogs in some other ciliate species, i.e., *E. caudatum*, *E. focardii*, *O. trifallax*, *Paramecium* spp., *Paraurostyla* sp., *P. persalinus, P. carnea*, *S. lemnae*, *S. stylifer*, *U. citrina*, *Urostyla* sp., *Tetrahymena* spp.

**FIGURE 2:**
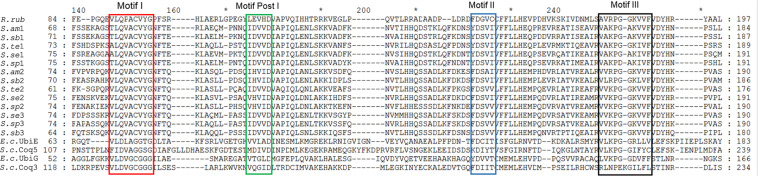
An alignment of *Spirostomum* and *Rhodospirillum* RquA with methyltransferases. The RquA sequences of *R. rubrum* (*R.rub*: ABC24022), *S. ambiguum* (*S.am*1, *S.am*2), *S. subtilis* (*S.sb*1, *S.sb*2, *S.sb*3), *S. teres* (*S.te*1, *S.te*2), *S. semivirscens* (*S.se*1, *S.se*2, *S.se*3) and *Spirostomum* sp., (*S.sp*1, *S.sp*2, *S.sp*3), were aligned with UbiE (YP026269) and UbiG (NP416735), Coq5 (CAY81708) and Coq3(CAY86194) of *Escherichia coli* (*E.c.*), and *Saccharomyces cerevisiae* (*S.c.*), respectively, using ClustalW. Four signature motifs (Motif I, Motif post-I, Motif II, and Motif III) in methyltransferases (indicated with boxes) were identified by using consensus motifs described previously (Petrossian and Clarke, 2009; Lonjers et al., 2012).

For comprehensive identification, all RquA sequences from *Spirostomum* spp., were compared with previously confirmed methyltransferases and analyzed for typical amino acid Motif-I of RquA (Lonjers et al., 2012). Protein sequence alignment showed that all RquA contained class I S-adenosyl methionine (SAM)-dependent domain and exhibited high similarity with methyltransferases within signature motifs (Motif I, Motif Post I, Motif II, Motif III); however, Motif-I in RquA sequences is distinct from other methyltransferases. The presence of glutamine and valine in Motif-I, is an identification feature in all RquA sequences instead of aspartate and glycine at their corresponding positions as in UbiE and UbiG sequences (Figure 2).

**FIGURE 3:**
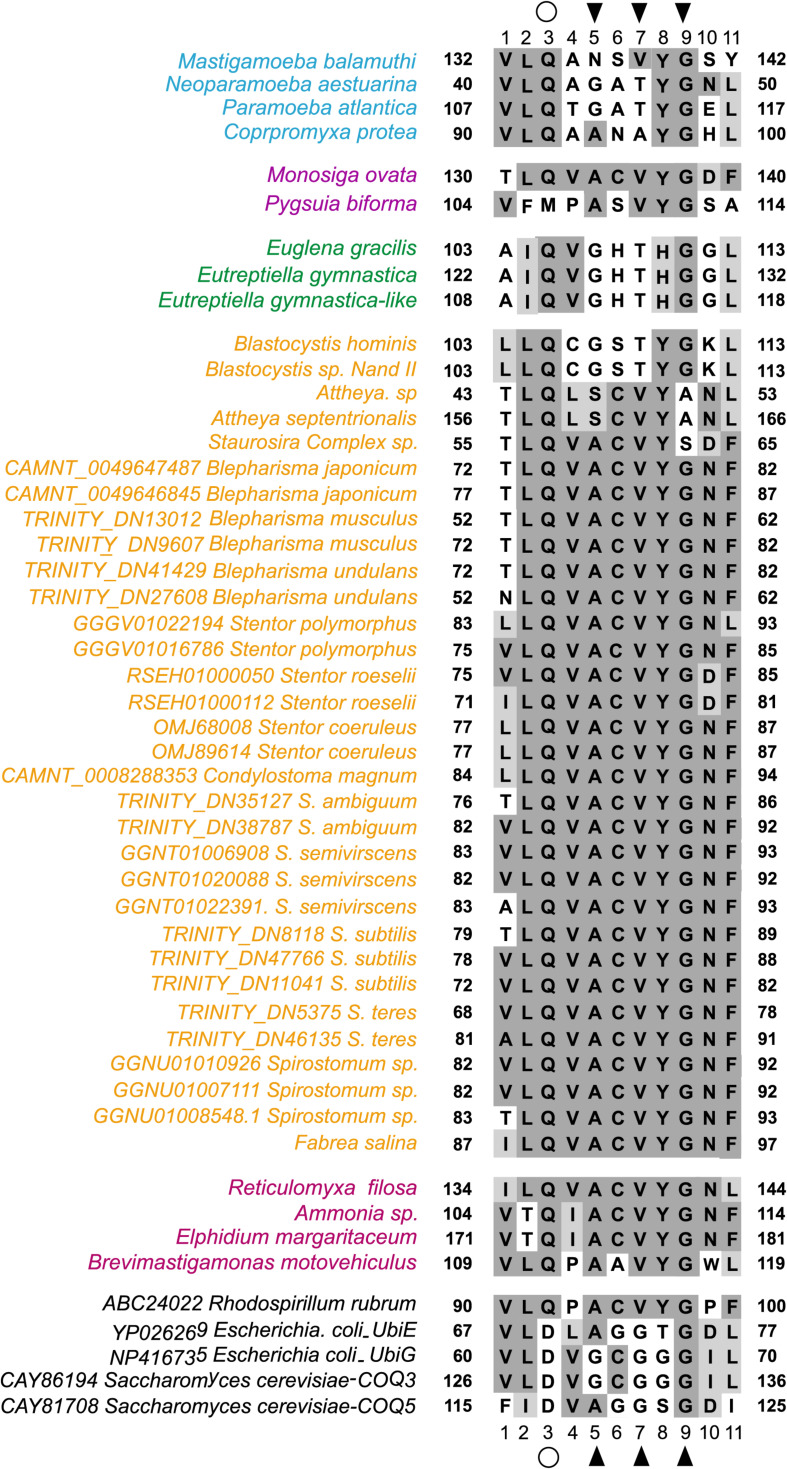
Comparison Motif-I between *Spirostomum* RquA orthologs and other S-adenosyl methionine (SAM) transferases. A substitution from aspartate to glutamine in RquA demonstrated with an open circle at position 3 and, contain a valine in place of glycine at position 7. The positions of the conserved residues that can interact with the carbonyl group of SAM are indicated with black triangles and taxa are colored according to group affiliation (Stairs et al., 2018). Different colors represents groups in Eukaryotes with RquA (blue, Amoebozoa; yellow, Stramenopiles and Alveolata; purple, Obazoa; green, Excavata; pink, Rhizaria). This figure is based on previously published data (Figure 2; Stairs et al., 2018) and is updated in this study.

In a detailed comparison of Motif-I within all eukaryotes RquA with other methyltransferases (UbiE/Coq5 and UbiG/Coq3), glutamine in place of aspartate or glutamate (position 3 in Figure 3), and valine in place of glycine (position 7 in Figure 3) could easily be identified. According Stairs et al. (2018), three conserved amino acid (CAA) residues within Motif-I, known to interact with the carbonyl group of SAM, could also be recognized in all eukaryotic RquA including *Spirostomum* spp. Thus, presence of Motif-I and its similarity (except some substitutions in Motif I) with others reported RquA, indicate that predicted sequences in this study are true RquA proteins and according to previous studies (Lonjers et al., 2012; Stairs et al., 2018).

Ubiquinone (Q) is an established precursor to RQ (rhodoquinone) in *R. rubrum* and usually produced in the mitochondrion in many anaerobic eukaryotes (Tran and Clarke, 2007). Therefore, we evaluated the presence of N-terminal mitochondrial targeting signals (MTS) in *Spirostomum* RquA homologs to confirm if these could show some affinity with mitochondria. Three independent tools, i.e., Target P (Emanuelsson et al., 2000), PSORT II (Nakao and Nakai, 2002), and Wolf PSORT (Horton et al., 2007), were used to predict the subcellular localization of candidate RquA proteins. At least two of these predictors programs indicated a MTS in the full-length and some partial RquA sequences from *S. ambiguum*, *S. subtilis*, and *S. tere*s. Presence of MTS in putative protein is accordance with previous studies that have predicted mitochondrial localization of RquA in anaerobic eukaryotes including *Spirostomum* species (Stairs et al., 2014; Hines et al., 2018). Results indicate that identified potential RquA sequences in the transcriptomes of *S. ambiguum*, *S. subtilis*, and *S. teres*, are indeed true homologs to RquA.

### Classification of RquA in the Class Heterotrichea

**FIGURE 4:**
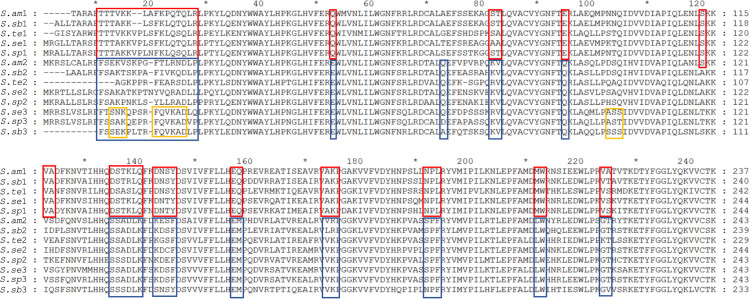
Alignment of all RquA identified orthologs from five species of genus *Spirostomum* showing conserved residues. Red and blue boxes indicate the locations, distribution, and differences of CAA (conserved amino acid) residues in the orthologs RquA type I (*S.am*1, *S.sub*1, *S.te*1, *S.se*1, *S.sp*1) and type II (*S.am*2, *S.sub*2, *S.te*2, *S.se*2, *S.sp*2, *S.sub*3, *S.se*3, *S.sp*3) in, *Spirostomum ambigumm* (*S.am*), *S. subtilis* (*S.sb*), *S. teres* (*S.te*), *S. semivirscens* (*S.se*), and *Spirostomum* sp. (*S.sp*), respectively. While, yellow box indicates conserved residues in RquA3 (*S.sb*3, *S.se*3, *S.sp*3).

Sequencing of transcriptome enabled the identification of (almost) all of the *Spirostomum* RquA proteins. The presence of CAA residues is an important contribution of structure/function relationships in a series of homologous proteins (Frieden, 2014). To confirm if the predicted RquA paralogs are highly conserved in *Spirostomum* spp., we carried out deep sequence analysis of *Spirostomum* RquA. In Figure 4, it is shown that 15 amino acid residues (other than methyltransferases motifs)/amino acid positions are highly conserved in all RquA sequences from five *Spirostomum* spp. On the basis of these conserved residual positions, *Spirostomum* RquA could be divided as; group-I includes five RquA1 (according to the nomenclature in this study) sequences; while, group-II includes RquA2 and RquA3. Based on sequence similarity, these two RquA groups are perhaps homologous, but show deviation from each other in characteristic ways (Figure 4); however, similar to RquA specific Motif region. Alignment results also showed that RquA3 are slightly different from RquA2 at three conserved residual positions within group-II. At this point, we can suggest that *Spirostomum* species have two to three *rquA* genes which can be classified into two types. All *rquA1* belongs to type-1 (= group-I), while *rquA2* and *rquA3* are of type-II. This classification can be revised in the future according to new data from *Spirostomum*.

**FIGURE 5:**
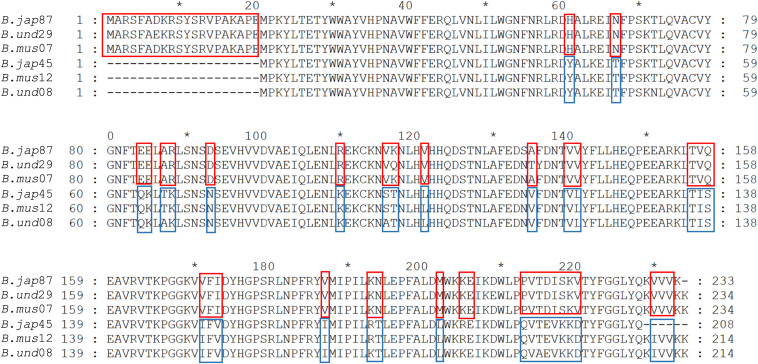
Alignment of RquA sequences from three *Blepharisma* species. Red and blue boxes indicate the locations, distribution, and differences of CAA residues in the RquA type-I [*B. jap*87 (CAMNT_0049647487); *B.und*29 (Trinity_DN41429); *B.mus*07 (Trinity_DN9607)] and type-II [*B.jap*45 (CAMNT_0049646845); *B.mus*12 (Trinity_DN13012); *B.und*08 (Trinity_DN27608)]. The “*B.jap*,” “*B.mus*,” and “*B.und*” represents *B. japonicum*, *B. musculus* and *B. undulans*, respectively.

Based on CAA residues, we analyzed RquA from *Blepharisma* and *Stentor* species separately and could also identified two RquA groups (Figure 5) in *Blepharisma* spp., while such sorting could not be predicted in *Stentor* species. Although, CAA residues distribution seemed more species-specific in the case of *Stentor* spp. RquA sequence analysis provides strong evidence that CAA residues in RquA paralogs are genus-specific and group oriented in *Blepharisma* and *Spirostomum* species.

### Validation of *rquA* Genes in *Spirostomum* Species

The *rquA* genes were amplified successfully using specific primers, and their partial sequences could only be determined. Genomic sequencing also confirmed two to three *rquA* genes in *S. ambiguum*, *S. teres*, and *S. subtilis*, respectively. Multiple alignment of the obtained genomic and transcriptomic sequences showed 99–100% similarity, and no intron region was found in the amplified partial genomic sequences.

### Occurrence of Quinone-Utilizing Enzymes in *Spirostomum* Species

**FIGURE 6:**
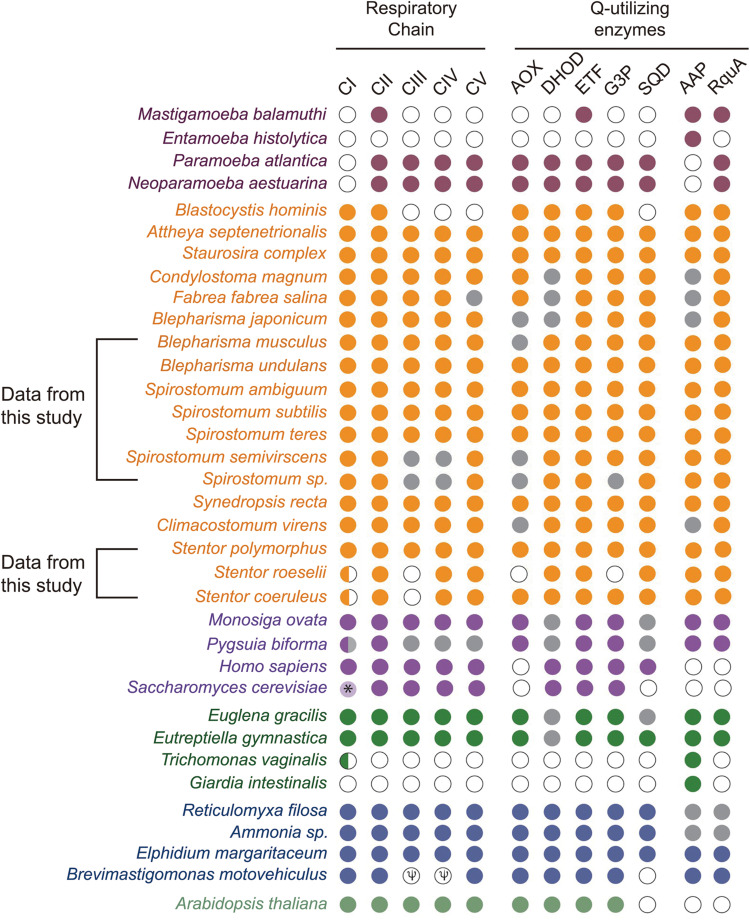
Co-occurrence and distribution of quinone-utilizing enzymes and RquA in the transcriptomes and genomes from Eukaryotes. This figure is based on previously published data (Figure 3; Stairs et al., 2018) and with additional information from *Spirostomum*, *Blepharisma* and *Stentor* spp., on respiratory chain elements (Complexes I–V, CI–CV), AOX (alternative oxidase), DHOH (dihydroorotate dehydrogenase), ETF (electron-transferring flavoprotein system components), G3P (glycerol-3-phosphate dehydrogenase, G3PDH), SQO (sulfite:quinone oxidoreductase), RquA, and AAP (indicates one or more anaerobiosis-associated proteins). Colored plots indicate presence and absences of proteins or protein subunits associated with anaerobic systems in Eukaryotes (maroon, Amoebozoa; yellow, Stramenopiles and Alveolata; mauve, Obazoa; green, Excavata; blue-gray, Rhizaria; dull-green, Archaeplastida). Gray and white circles indicate no homologs of above mentioned proteins in transcriptome and genome data, respectively; while, half circle indicates the presence of only two subunits (NUOE and NUOF) in CI complex; while “Ψ” indicates pseudogenes in the species.

Presence of RquA in the transcriptomes of *Spirostomum* and *Blepharisma*, as well as in the genome of *Stentor* species, indicates that these organisms could also encode at least quinone-reducing and re-oxidizing enzymes (Stairs et al., 2018). Based on detail search, we could identified quinone-utilizing enzymes (Supplementary Table S2) such as CI and CIII (respiratory complexes); quinone biosynthesis enzymes (these include COQ1–7); AOX (alternative oxidase); DHOD (dihydroorotate dehydrogenase); electron transferring flavoprotein (ETF) system [contains ETFa and b; ETF dehydrogenase (ETFDH)]; G3PDH (glycerol-3-phosphate dehydrogenase); and SQO (sulfide:quinone oxidoreductase). Blast results showed that *Spirostomum* as well as *Blepharisma*, and *Stentor* spp., have at least CII and ETF along with a minimum of four quinone-utilizing enzymes. Figure 6 (we have modified figure 3 from Stairs et al., 2018) provides an update information on quinone-utilizing enzymes and RquA in *Blepharisma*, *Spirostomum*, and *Stentor* species including other *rquA*-containing eukaryotes.

### Phylogenetic Analysis of RquA

For the confirmation of the relationship between obtained RquA sequences from *Spirostomum* spp., (in this study), a phylogenetic analysis was carried out with reported RquA sequences from Eukaryotes (Stairs et al., 2018) including additional identified RquA sequences obtained from the published transcriptomes [*Spirostomum* spp., *Stentor polymorphus* (Hines et al., 2018; Onsbring et al., 2018)] and genome of *S. roeselii* (PRJNA507905). We have also included additional RquA sequences from the transcriptome of *B. undulans* (SRR12647630) and *B. musculus* (SRR12647629). Results showed that members of the class Heterotrichea formed a separate monophyletic cluster of the RquA protein from eukaryotic species. The phylogenetic position of RquA of *Spirostomum* spp., within RquA clade of the heterotrichs, support to that the all identified RquA proteins in this study are true RquA.

**FIGURE 7:**
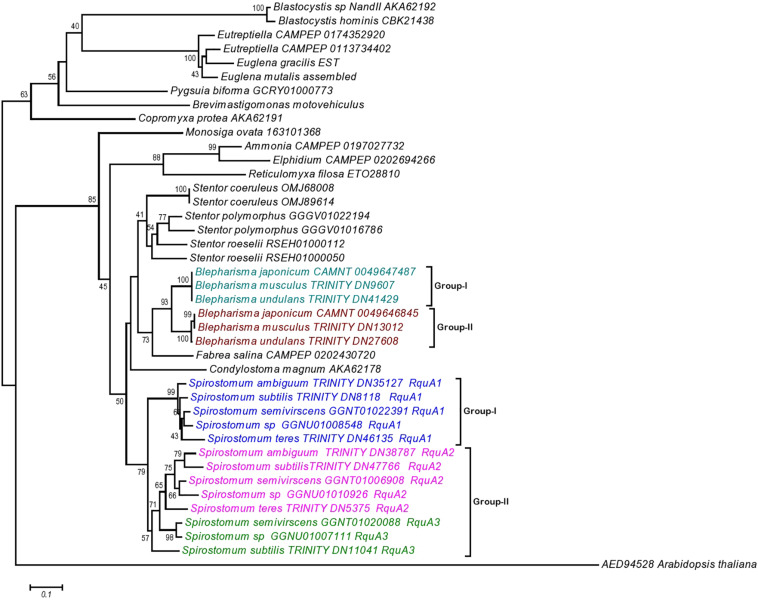
The Neighbor-Joining (NJ) phylogenetic tree of ortholog RquA proteins from eukaryotes and *Spirostomum* species. HD gene (AED94528) of *Arabidopsis thaliana* is taken as out group in this tree. RquA proteins from *Spirostomum* are classified as RquA1, RquA2, and RquA3 that belong to group-I and II. Group-I contains RquA1 of five different *Spirostomum* species, whereas group-II represents RquA2 and RquA3 collectively.

The phylogenetic analysis also showed that the RquA from *Spirostomum* spp., could be divided into two subclades (termed as I and II), each clade comprising 5–8 paralogs from *Spirostomum* spp. (Figure 7). The subclade-I comprise five all newly identified RquA1 of *S. semivirscens* (GGNT01022391), *Spirostomum* sp., (GGNU0101926), *S. ambiguum* (TRINITY_DN35127), *S. subtilis* (TRINITY_DN8118), and *S. teres* (TRINITY_DN46135). However the majority of RquA belongs to subclade-II, which includes two homologs of *S. subtilis* (*S.sb*-RquA2, *S.sb*-RquA3), *S. semivirscens* [(GGNT01020088 = *S.sm*-RquA2), (GGNT0106908 = *S.sm*-RquA3)] and *Spirostomum sp.* [(GGNU01007111 = *S.sp*-RquA2), (GNU01010926 = *S.sp*-RquA3)], while one from *S. ambiguum* (*S.am*-RquA2) and *S. teres* (*S.te*-RquA2). However, RquA3 of *S. semivirscens, Spirostomum sp.*, and *S. subtilis* showed a close affinity and positioned separately within subclade-II. Similarly, RquA paralogs from three species of *Blepharisma* could be divided into the two subclades (Figure 7) and also support RquA groups based on CAA residues in this genus.

The presence of RquA-type-I (= all RquA1) and type-II (= all RquA2 and RquA3) in *Spirostomum* spp., strongly suggests that at least one of each type-I and type-II *rquA* gene existed already in the last common ancestor of ciliates or whether one of these two types of RquA group represents a more ancestral type is not known. We have included potential RquA [GGGV01016786 = identified as new potential RquA in this study); GGGV01022194 = previously reported by Stairs et al. (2014) and Hines et al. (2018)] from the sequences of *S. polymorphus* (PRJNA433765), *S. roeselii* [(RSEH0100050, RSEH01000112) BioProject: PRJNA507905], *Blepharisma undulans* (Trinity_DN27608, Trinity_DN41429), *Blepharisma musculus* (Trinity_DN13012; Trinity_DN9607), and *Stentor coeruleus* (OMJ89614; OMJ68008) in the phylogenetic analysis as an evidence that at least two *rquA* paralogs exists in some members of the class Heterotrichea. In the case of *Blepharisma japonicum* two RquA sequences (CAMNT_0049647487, CAMNT_0049646845) have already been mentioned (Stairs et al., 2014; Hines et al., 2018) that support our results.

### Detection of RQ and Q in Lipid Extracts

To establish a relationship of *rquA* in RQ synthesis in cell and RquA expression in transcriptomes of *Spirostomum* and *Blepharisma* species, 15 days old adherent cell cultures grown in plates were analyzed for rhodoquinone and ubiquinone (i.e., RQ_10_ and Q_10_) synthesis *in vivo* using LC-MS. Relative high COD (chemical oxygen demand) in adherent cell cultures of *Spirostomum* and *Blepharisma* species supported that cells actually experienced anaerobic condition. The LC-MS results showed no RQ_10_ biosynthesis, but could detect Q_10_ in the cells of *Spirostomum* and *Blepharisma* species (Supplementary Figure S3).

Previously, Q_10_ is a reported biosynthetic precursor of RQ_10_ (although it was not directly confirmed using radiolabeled Q_10_), and specific activity of RQ_10_ may decline in the culture with the time (Parson and Rudney, 1965; Brajcich et al., 2010). It is also possible that RQ_10_ was not detected in the current study due to degradation, low quantity, or lack of sensitivity of the analysis method. We have also analyzed the lipid contents of prey prokaryotes cultures (grown without *Spirostomum* or *Blepharisma* spp.) and found no indication of RQ_10_ and Q_10_, suggesting that prokaryotes are not the exogenous source of Q_10_, but *Spirostomum/Blepharisma* spp., can synthesize it.

## Discussion

We have collected *Spirostomum* species from freshwater habitat from a pond in Fuzhou, China. The isolates used in this study represent a new record for Fuzhou and their further habitat in China. Recently *Spirostomum minus, S. subtilis*, and *S. teres* have also been recorded from the surface layer of lake-bed sediment in Zhongshan Park, Qingdao, China (Chi et al., 2020). On the basis of morphological features of genus *Spirostomum* like uniformly ciliated body that may be either cylindrical or flattened, collected species were identified as *S. ambiguum*, *S. subtilis*, and *S. teres*. The molecular analysis also revealed variable SSUrDNA sequence between *S. ambiguum*, *S. subtilis*, and *S. teres* populations, confirming the identification. The distribution of *S. ambiguum*, *S. subtilis*, and *S. teres* has been expanded between the sampling sites investigated before in China or other locations globally supports that microbial species maintain their population in suitable conditions (Finlay, 2002; Hines et al., 2016). In phylogenetic tree *S. ambiguum* and *S. subtilis* clades were positioned differently as compared to previous studies (Boscaro et al., 2014; Shazib et al., 2014). However, tree topology results were similar to Chi et al. (2020) with bootstrap support for *S. ambiguum* together with *S. subtilis* clade in the genus *Spirostomum*.

The RquA found in the transcriptomes of three *Spirostomum* spp., showed homology to related members of class Heterotrichea (Hines et al., 2018). Topology tests also indicate that RquA clade divides as subclade-I and II in *Spirostomum* species, strongly support the existence of multiple *rquA* genes in the genomes *Spirostomum* spp. The presence of RquA paralogs also suggests that these organisms could have the maximum ability to produce and utilize RQ in the response and adaptation to anaerobic conditions. In many eukaryotes, RQ biosynthesis and fumarate usage as a terminal acceptor in the electron transport chain in the mitochondria, is an adaptive feature under low-oxygen conditions (Stairs et al., 2018).

Similar to many Q-utilizing enzymes, the RQ transports electron between complex I and complex II (Ma et al., 1993), therefore presence of other quinone-utilizing complexes, also predict the role of RquA in RQ synthesis. On the basis of these facts, we searched RQ-utilizing enzymes that reduce and re-oxidize it. The presence of other Q-utilizing complexes in the transcriptomes of *Spirostomum* spp., indicates quinone utilization (Figure 6 and Supplementary Table S2). According to the aerobic model, the Q-utilizing systems are located in the mitochondria of many eukaryotes (Marreiros et al., 2016). A recent study also shows that all RquA-containing eukaryotes including some protists i.e., *Blastocystis, Mastigamoeba*, and *Pygsuia* with mitochondrion-related organelles (MROs), have up to four other quinone-utilizing complexes and enzymes; while, in many eukaryotes with Q-biosynthesis pathway, RquA coding could not be identified (Stairs et al., 2018).

Presences of four distinct and characteristic motifs of Class I SAM methyltransferases, could also be identified in RquA from *Spirostomum* spp. These motifs are important for protein folding and SAM binding (Petrossian and Clarke, 2009). Further analysis showed that key SAM binding sites in Motif I (Figure 3) is similar in the bacterial and eukaryotic RquA (Lonjers et al., 2012; Stairs et al., 2018) and support the identification of new RquA in *Spirostomum* spp. However, split in RquA clade of *Spirostomum* and *Blepharisma* species (Figure 7) between two distinct groups (I and II) composed strengthen the presences of multiple *rquA* (Figure 7). Sequences survey in the transcriptomes and genomes of *Stentor* and *Blepharisma* species also provides an evidence that at least two *rquA* can be present in ciliates (Stairs et al., 2014; Hines et al., 2018).

Presence of *rquA* in the *Spirostomum* spp., as well as in other members of class Heterotrichea, suggests that the RQ biosynthesis pathway of these ciliates is associated to the RquA-based system and these organisms have the capacity to synthesize and utilize RQ in adaptation to hypoxic conditions. RQ biosynthesis has been detected in many eukaryotes [*Caenorhabditis elegans* (Takamiya et al., 1999); *Ascaris suum* (Kita et al., 1988); *Fasciola hepatica* (Van Hellemond et al., 1996)] including protists [*Euglena gracilis* (Castro-Guerrero et al., 2005); *Nyctotherus ovalis* (Boxma et al., 2005); *Pygsuia biforma* (Stairs et al., 2018)] so far; however, it is still not confirmed in many other anaerobic eukaryotes. We have also not found any direct indication of RQ_10_ synthesis in the *Spirostomum* and *Blepharisma* spp., but only the presence of Q_10_. Previously, the specific activity of both Q_10_ and RQ_10_ has been measured in anaerobically grown *R. rubrum* and suggest Q_10_ a precursor of RQ_10_ (Brajcich et al., 2010).

Based on the sequence identity to RquA found in *R. rubrum* and *Spirostomum* spp., we suggest that *S. ambiguum*, *S. subtilis*, and *S. teres* use rhodoquinol-dependent fumarate reduction to respire under anaerobic conditions. The presence of more than one type of RquA suggested that *Spirostomum* spp., have multiple *rquA* and putative all *rquA* genes or only one in *Spirostomum* species might be involved in the pathway of anaerobic respiration. Monophyletic group of all heterotrichs species in a phylogenetic tree of RquA proteins, is also constant in the topology of their respective SSU rDNA genes (Supplementary Figure S2) and support RquA hierarchy in their genomes/transcriptomes. Further characterization of *rquA* genes with the reference to their specific functions, is currently under way in our laboratory. Complete information of functional differences among these *rquA* paralogs can provide essential understanding of anaerobic adaptation in eukaryotes.

## Conclusion

In this study transcriptomic data has been generated in the effort to know more about the anaerobic existence of the *Spirostomum* genus. Transcriptomes analysis suggests that *S. ambiguum*, *S. subtilis*, and *S. teres* can switch to RQ-dependent pathway under anaerobic conditions like other protists that also thrive in anoxic habitats. With the rDNA data, at least two to three *rquA* genes can be assigned to *S. ambiguum*, *S. subtilis*, and *S. teres*. We also suggest that more than one RquA in *Spirostomum* species could have different functions like, (1) one RquA will be functional while other remains non-functional; (2) one or many RquA adopt a novel function (neo-functionalization); or sub-functional (required at least two genes for normal function).

## Data Availability Statement

Raw sequences have been deposited to NCBI Short Read Archive (SRA) database (http://www.ncbi.nlm.nih.gov/Traces/sra/) for *S. ambiguum* (SRX8646480), *S. subtilis* (SRX8646481), *S. teres* (SRX8646482), B. musculus (SRR12647629), and B. undulans (SRR12647630).

## Author Contributions

IM and JC conceived the project. IM, SW, and SRW collected the samples and did experimental protocol to identify and confirmation of *rquA* genes. RC, YC, and CL were involved in data acquisition and performed the analyses. IM drafted the manuscript. All authors have contributed to modification and approved the final version of the manuscript.

## Conflict of Interest

The authors declare that the research was conducted in the absence of any commercial or financial relationships that could be construed as a potential conflict of interest.

## Footnotes

1 http://www.phylogeny.fr/index.cgi

2 https://code.google.com/p/corsetproject/

3 http://www.ncbi.nlm.nih.gov/Traces/sra/

## Supplementary Material

Supplementary Figure 1 Geographical location of Fuzhou city and photographs of the sampling sites in Fujian Agriculture and Forestry University (FAFU) campus. **(A)** partial map of Fujian province showing location of Fuzhou city; **(B)** portion of FAFU map showing location of a fresh water pond site (indicated with red dot); **(C)** habitat of sampling site on the bank of pond; **(D)** sampling points (indicated with red arrow heads) for *Spirostomum* and *Blepharisma* species on the bank of fresh water pond in FAFU campus.

Supplementary Figure 2 Phylogenetic tree is constructed using NJ method from an alignment of SSU sequences from closely related heterotrichs. Topology of SSU rDNA genes in heterotrichs also support the phylogenetic position of RquA proteins in Figure 7.

Supplementary Figure 3 Mass spectra of Q_10_ and RQ_10_ in *Spirostomum*, *Blepharisma* species and prey bacteria. All spectra were obtained using a Thermo QE as specified in section “Materials and Methods.”

Supplementary Table 1 List of the primers used in this study for partial identification of *rquA* genes in *Spirostomum* species based on transcriptome data.

Supplementary Table 2 Co-occurrence of RquA with quinone-utilizing enzymes.
