# Supplementary figures and images for: Transcriptome Profiling Revealed Multiple rquA Genes in the Species of Spirostomum (Protozoa: Ciliophora: Heterotrichea)
Source: Front Microbiol. 2021 Jan 5;11:574285. doi: 10.3389/fmicb.2020.574285 (PMC7813818; doi:10.3389/fmicb.2020.574285)

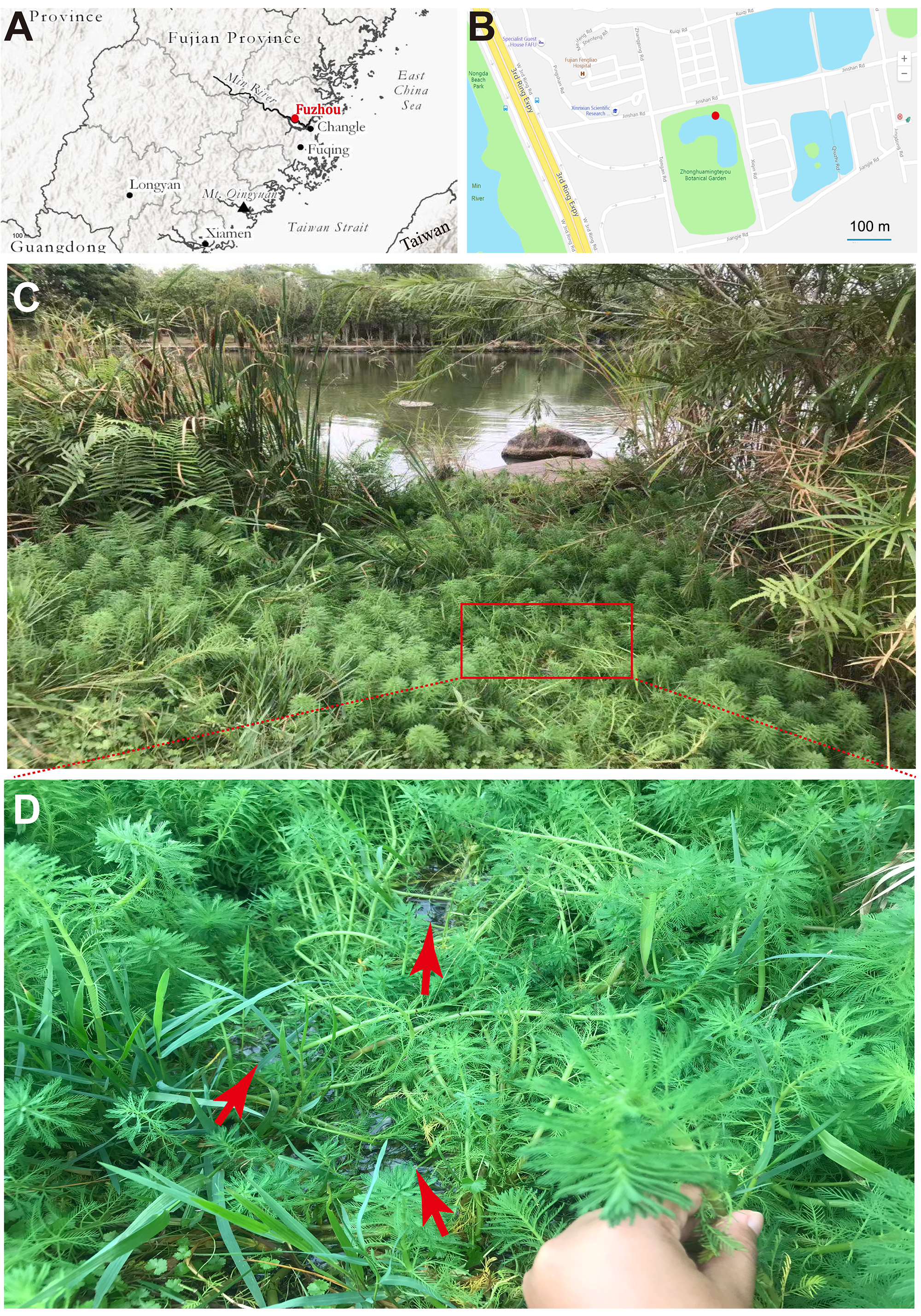

Supplement: Supplementary Figure 1 — Geographical location of Fuzhou city and photographs of the sampling sites in Fujian Agriculture and Forestry University (FAFU) campus. (A) partial map of Fujian province showing location of Fuzhou city; (B) portion of FAFU map showing location of a fresh water pond site (indicated with red dot); (C) habitat of sampling site on the bank of pond; (D) sampling points (indicated with red arrow heads) for Spirostomum and Blepharisma species on the bank of fresh water pond in FAFU campus. [file Image_1.JPEG]

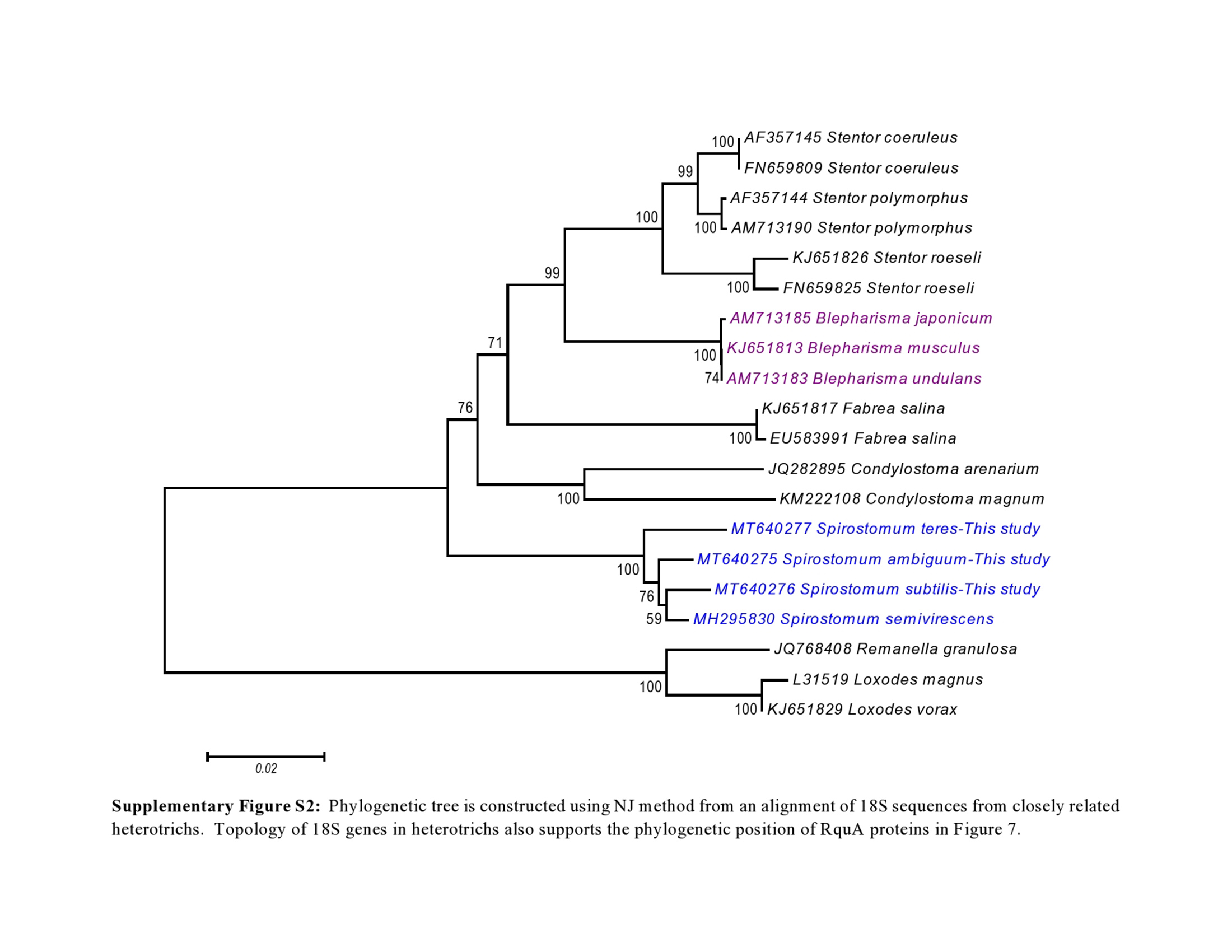

Supplement: Supplementary Figure 2 — Phylogenetic tree is constructed using NJ method from an alignment of SSU sequences from closely related heterotrichs. Topology of SSU rDNA genes in heterotrichs also support the phylogenetic position of RquA proteins in Figure 7. [file Image_2.JPEG]

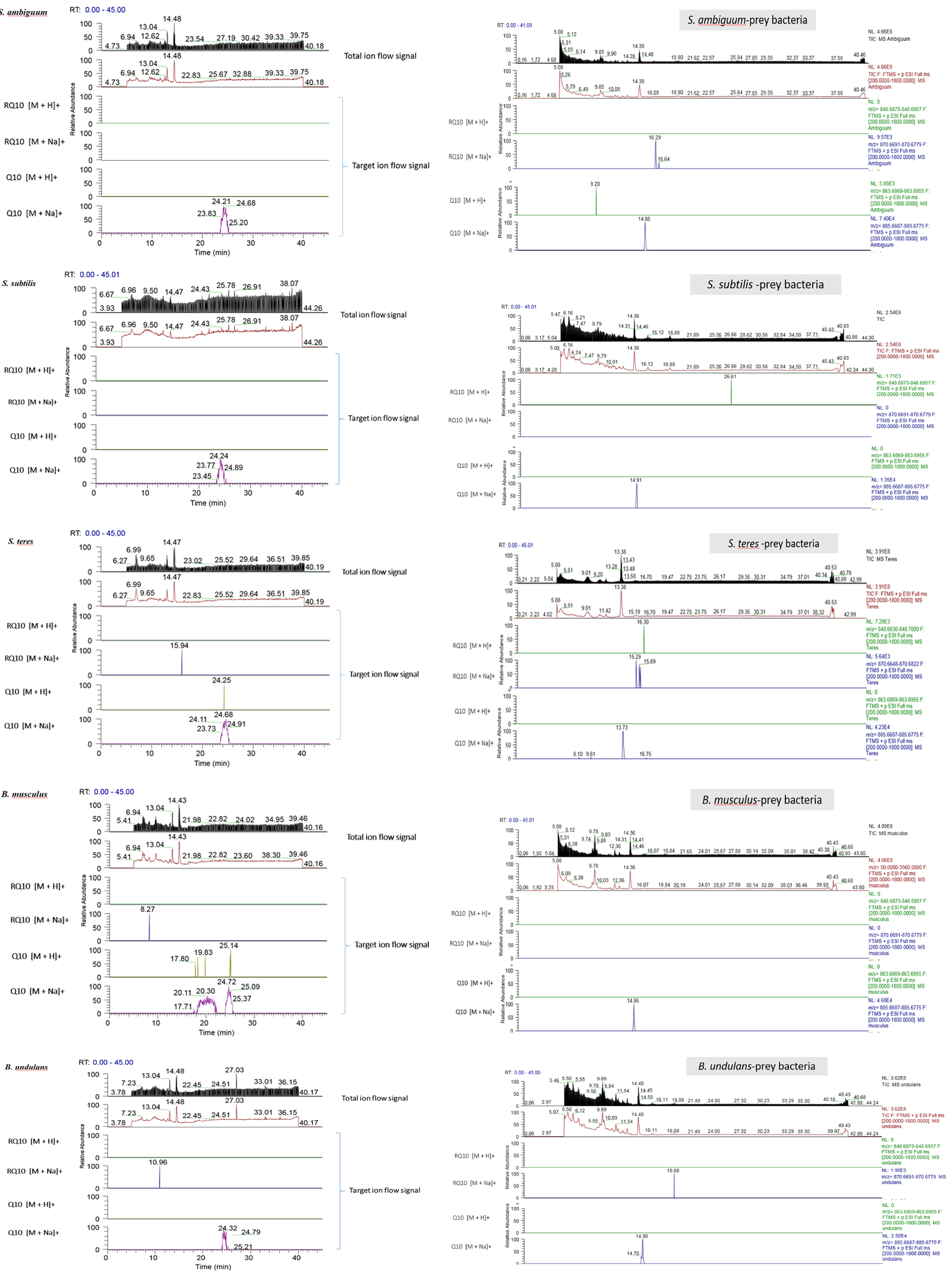

Supplement: Supplementary Figure 3 — Mass spectra of Q10 and RQ10 in Spirostomum, Blepharisma species and prey bacteria. All spectra were obtained using a Thermo QE as specified in section “Materials and Methods.” [file Image_3.TIF]
