# Supplementary material for: Transcriptome Profiling Revealed Multiple rquA Genes in the Species of Spirostomum (Protozoa: Ciliophora: Heterotrichea)
Source: Front Microbiol. 2021 Jan 5;11:574285. doi: 10.3389/fmicb.2020.574285 (PMC7813818; doi:10.3389/fmicb.2020.574285)
Supplement: Supplementary Table 1 — List of the primers used in this study for partial identification of rquA genes in Spirostomum species based on transcriptome data. [file Table_1.docx]

**Supplementary Table S1:** List of the primers used in this study for partial identification of *rquA* genes in *Spirostomum* species based on transcriptome data

| **Species** | **Gene** | **Primer sequence (5′-3′)** | **Tm (°C)** | **Obtained Amplicon size (bp)** |
| --- | --- | --- | --- | --- |
| *S. ambiguum* | *S.am-rquA1* (TRINITY_DN35127) | F: CACGGCTCGCGCTTTCAC | 63 | 710 |
|  |  | R: ACTTCGTGCAGACGACCTTC | 63 |  |
|  | *S.am-rquA*2  (TRINITY_DN38787) | F:GCGGAAATATGGCCGATAATG | 61 | 740 |
|  |  | R: AGCAAACGACTTTCTGGTAGAG | 62 |  |
| *S. subtilis* | *S.sb*-*rquA*1 (TRINITY_DN8118) | F: GGGCACTCCTCACAGCTC | 60 | 740 |
|  |  | R: TACTTCGTGCACACGACCTTC | 60 |  |
|  | *S.sb*-*rquA*2 (TRINITY_DN47766) | F: GCAGCCCTTCGTTTCTTTTC | 62 | 680 |
|  |  | R: ACTGCAAACGACTTTTTGATAG | 59 |  |
|  | *S.sb*-*rquA*3 (TRINITY_DN11041) | F: GAAAAACCTCTGACGCGCTTTC | 62 | 670 |
|  |  | R: AGTGCAGACAAGCTTCTGGTAG | 62 |  |
| *S. teres* | *S.te-rquA*1 (TRINITY_DN46135) | F: TCTCTTATGCTCGCTGGTTC | 61 | 730 |
|  |  | R: ACTTAGTGCAGACCATCTTT | 62 |  |
|  | *S.te*-*rquA*2 (TRINITY_DN5375) | F: CAAGCCTCCTCGCTTTGAAG | 60 | 690 |
|  |  | R:AGTGCAGACTACTTTCTGGTAG | 60 |  |
