# Supplementary material for: Transcriptome Profiling Revealed Multiple rquA Genes in the Species of Spirostomum (Protozoa: Ciliophora: Heterotrichea)
Source: Front Microbiol. 2021 Jan 5;11:574285. doi: 10.3389/fmicb.2020.574285 (PMC7813818; doi:10.3389/fmicb.2020.574285)
Supplement: Supplementary Table 2 — Co-occurrence of RquA with quinone-utilizing enzymes. [file Table_2.docx]

**Supplementary Table S2.** **Co-occurrence of RquA with quinone-utilizing enzymes.** Transcriptomes and genomes of *Spirostomum* spp., (*S. ambiguum* = *S.* *sam*; *S. subtilis* =*S. sub*; *S. teres = S. ter*; *S. semivirscens* = *S. sem*; *Spirostomum* sp = *S. sp*), *Blepharisma* spp., (*B. musculus* = *B. mus*; *B. undulans* = *B. und*) and *Stentor* spp., (*St. coeruleus = St. coe; St.* *polymorphus*= *St. pol* ; *St. roeselii* = *St. roe*), were searched for the presence of additional respiratory chains with related enzymes according to Stairs et al. (2018). The plus (+) sign indicates presence of respective component /enzyme while other signs (?/-) indicates not detected in transcriptome and genome respectively.

|  | **Protein Name** | **Abbreviation** | ***S. amb*** | ***S. sub*** | ***S. ter*** | ***S.*** ***sem*** | ***S. sp*** | ***B. mus*** | ***B. und*** | ***St. coe*** | ***St. pol*** | ***St. roe*** |
| --- | --- | --- | --- | --- | --- | --- | --- | --- | --- | --- | --- | --- |
| **Q-utilizing enzymes** | Succinate dehydrogenase flavoprotein subunit (Complex II) | SDHA (CII) | **+** | **+** | **+** | **+** | **+** | **+** | **+** | **+** | **+** | **+** |
|  | Succinate dehydrogenase Fe-S cluster subunit (Complex II) | SDHB (CII) | **+** | **+** | **+** | **+** | **+** | **+** | **+** | **+** | **+** | **+** |
|  | Succinate dehydrogenase large cytochrome b subunit (Complex II) | SDHC (CII) | **?** | **?** | **?** | **?** | **?** | **?** | **?** | **-** | **?** | **-** |
|  | Succinate dehydrogenase small cytochrome b subunit (Complex II) | SDHD (CII) | **?** | **?** | **?** | **?** | **?** | **?** | **?** | **-** | **?** | **-** |
|  | Ubiquinole:cytochrome c oxidoreductase | CIII | **+** | **+** | **+** | **?** | **?** | **+** | **+** | **-** | **+** | **-** |
|  | Alternative oxidase | AOX | **+** | **+** | **+** | **?** | **?** | **?** | **+** | **-** | **+** | **-** |
|  | Glycerol-3-Phosphate dehydrogenase | G3PDH | **+** | **+** | **+** | **+** | **?** | **+** | **+** | **+** | **+** | **-** |
|  | Electron transfering flavoprotein dehydrogenase | ETFDH | **+** | **+** | **+** | **+** | **+** | **+** | **+** | **+** | **+** | **+** |
|  | Electron transfering flavoprotein alpha | ETFa | **+** | **+** | **+** | **+** | **+** | **+** | **+** | **+** | **+** | **+** |
|  | Electron transfering flavoprotein beta | ETFb | **+** | **+** | **+** | **+** | **+** | **+** | **+** | **+** | **+** | **+** |
|  | NADH:Ubiquinone oxidoreductase (Complex I) | CI | **+** | **+** | **+** | **+** | **+** | **+** | **+** | **-** | **+** | **-** |
|  | NADH:Ubiquinone oxidoreductase E subunit (Complex I) | NuoE | **+** | **+** | **+** | **+** | **+** | **+** | **+** | **+** | **+** | **+** |
|  | NADH:Ubiquinone oxidoreductase F subunit (Complex I) | NuoF | **+** | **+** | **+** | **+** | **+** | **+** | **+** | **+** | **+** | **+** |
|  | Dihydroorotate dehydrogenase (Q-dependent) | DHOH | **+** | **+** | **+** | **+** | **?** | **+** | **+** | **+** | **+** | **+** |
|  | sulfide:quinone oxidoreductase | SQO | **+** | **+** | **+** | **+** | **+** | **+** | **+** | **+** | **+** | **+** |
|  |  |  |  |  |  |  |  |  |  |  |  |  |
| **UQ biosynthesis** | Hexaprenyl pyrophosphate synthetase (EC 2.5.1.83) | COQ1 | **+** | **+** | **+** | **+** | **+** | **+** | **+** | **+** | **+** | **+** |
|  | 4-hydroxybenzoate octaprenyl transferase (EC:2.5.1.39) | COQ2 | **+** | **+** | **+** | **+** | **+** | **+** | **+** | **+** | **+** | **+** |
|  | hexaprenyldihydroxybenzoate methyl transferase (EC:2.1.1.64 2.1.1.114) | COQ3 | **+** | **+** | **+** | **+** | **?** | **+** | **+** | **+** | **+** | **+** |
|  | N, N-dimethylaniline monooxygenase COQ6 (EC: 1.14.13.-) | COQ6 | **+** | **+** | **+** | **?** | **?** | **+** | **+** | **+** | **+** | **-** |
|  | 2-hexaprenyl-6-methoxy-1,4-benzoquinone methyl transferase (EC:2.1.1.201) | COQ5 | **+** | **+** | **+** | **+** | **+** | **+** | **+** | **+** | **+** | **+** |
|  | Monooxygenase CAT5 (EC: 1.14.13.-) | COQ7 | **+** | **+** | **+** | **+** | **+** | **+** | **+** | **+** | **+** | **+** |
|  | Phenylacrylic acid decarboxylase PAD1 | PAD1 | **+** | **?** | **+** | **?** | **?** | **?** | **?** | **-** | **?** | **-** |
|  |  |  |  |  |  |  |  |  |  |  |  |  |
| **Energy generation enzymes** | Cytochrome c oxidase | CIV | **+** | **+** | **+** | **?** | **?** | **+** | **+** | **-** | **?** | **-** |
|  | ATP synthase | CV | **+** | **+** | **+** | **+** | **+** | **+** | **+** | **+** | **+** | **+** |
|  | Succinyl-CoA synthetase alpha subunit | SCSa | **+** | **+** | **+** | **+** | **+** | **+** | **+** | **+** | **+** | **+** |
|  | Succinyl-CoA synthetase beta subunit | SCSb | **+** | **+** | **+** | **+** | **+** | **+** | **+** | **+** | **+** | **+** |
|  | Acetyl:succinyl CoA transferase Subtype 1A | ASCT 1A | **?** | **?** | **?** | **?** | **?** | **?** | **?** | **-** | **?** | **-** |
|  | Acetyl:succinyl CoA transferase Subtype 1B | ASCT 1B | **+** | **+** | **?** | **?** | **?** | **+** | **+** | **-** | **+** | **-** |
|  | Acetyl:succinyl CoA transferase Subtype 1C | ASCT 1C | **?** | **?** | **+** | **?** | **?** | **?** | **+** | **-** | **?** | **-** |
|  | Acetyl-CoA synthetase | ACS | **+** | **?** | **?** | **?** | **+** | **?** | **+** | **+** | **?** | **-** |
|  | Fumarate hydratase Class II | FUM Class II | **+** | **+** | **+** | **+** | **+** | **+** | **+** | **+** | **+** | **+** |
|  |  |  |  |  |  |  |  |  |  |  |  |  |
| **Anaerobiosis- Associated Proteins (AAP)** | Rhodoquinone biosynthesis protein | RQUA | **+** | **+** | **+** | **+** | **+** | **+** | **+** | **+** | **+** | **+** |
|  | Fumarate hydratase Class I (Anaerobic) | FUM Class I | **+** | **+** | **+** | **+** | **?** | **?** | **+** | **-** | **?** | **-** |
|  | [FeFe] Hydrogenase | HydA | **+** | **+** | **+** | **+** | **+** | **+** | **+** | **+** | **+** | **+** |
|  | Pyruvate:ferredoxin oxidoreductase | PFO | **+** | **+** | **+** | **?** | **+** | **?** | **?** | **-** | **+** | **-** |
|  | Hydrogenase maturase protein E | HydE | **+** | **?** | **+** | **+** | **+** | **+** | **+** | **+** | **+** | **+** |
|  | Hydrogenase maturase protein F | HydF | **+** | **?** | **?** | **?** | **?** | **?** | **+** | **+** | **+** | **+** |
|  | Hydrogenase maturase protein G | HydG | **+** | **+** | **+** | **?** | **+** | **?** | **?** | **-** | **?** | **-** |
